# The Foliar Endophyte *Phialocephala scopiformis* DAOMC 229536 Proteome When Grown on Wood Used as the Sole Carbon Source

**DOI:** 10.1128/MRA.01280-18

**Published:** 2019-02-07

**Authors:** Jennifer M. Bhatnagar, Grzegorz Sabat, Daniel Cullen

**Affiliations:** a Department of Biology, Boston University, Boston, Massachusetts, USA; b University of Wisconsin—Madison Biotechnology Center, Madison, Wisconsin, USA; c USDA, Forest Products Laboratory, Madison, Wisconsin, USA; University of California, Riverside

## Abstract

The conifer needle endophyte Phialocephala scopiformis DAOMC 229536 was cultivated in medium containing ground Pinus contorta wood as the sole carbon source. Mass spectrometry analyses identified 590 proteins. The expression of extracellular hydrolases and oxidoreductases indicates a capacity to degrade wood. The results clearly demonstrate the latent saprophytic potential of P. scopiformis.

## Abstract (precis)

The conifer needle endophyte Phialocephala scopiformis DAOMC 229536 was cultivated in medium containing ground Pinus contorta wood as the sole carbon source. Mass spectrometry analyses identified 590 proteins.

## ANNOUNCEMENT

Metatranscriptomes of decayed Pinus contorta logs (1) have shown that transcripts closely related to Phialocephala scopiformis, an endophyte of conifer needles, are the most abundant ascomycete representatives. Here, we show that P. scopiformis DAOMC 229536, whose genome was previously sequenced, is capable of utilizing P. contorta wood as a sole carbon source and, in doing so, produces an array of hydrolytic and oxidative enzymes.

Two-liter flasks containing 250 ml of basal salt medium (2) were supplemented with 1.25 g of ground P. contorta wood as the sole carbon source. The medium was inoculated with P. scopiformis DAOMC 229536 (Canadian Collection of Fungal Cultures) and placed on a rotary shaker (150 rpm). After 5 and 7 days of incubation at 22 to 24°C, the cultures were filtered successively through Miracloth (Calbiochem) and Whatman filter papers #50 and #541. The filtrate proteins were precipitated with 10% (wt/vol) trichloroacetic acid and washed three times in cold acetone before air drying. Total proteins from the pellets were further purified via methanol-chloroform-water partitioning, where chloroform and methanol were added to pellets first, followed by water, and these were allowed to partition, with a protein interphase formed between the polar and nonpolar fractions. After multiple methanol washes, these finely purified protein preps were ultimately resolubilized in 8 M urea–50 mM NH_4_HCO_3_ (pH 8.5)–1 mM Tris-HCl.

Nano-liquid chromatography–tandem mass spectrometry (nanoLC-MS/MS) was used to identify proteins (1, 3–5). Equal amounts of total protein per sample were trypsin/LysC digested and OMIX C_18_ solid-phase extraction (SPE) purified (Agilent Technologies); and finally, 2 µg was loaded for nanoLC-MS/MS analysis using an Agilent 1100 nanoflow system connected to a hybrid linear ion trap-orbitrap mass spectrometer (LTQ-Orbitrap Elite; Thermo Fisher Scientific) equipped with an EASY-Spray electrospray source. Chromatography of peptides prior to mass spectral analysis was accomplished using a capillary emitter column (PepMap C_18_, 3 µM, 100 Å, 150 by 0.075 mm; Thermo Fisher Scientific), onto which 2 µl of purified peptides was automatically loaded. The nano-high-performance liquid chromatography (NanoHPLC) system delivered solvents A (0.1% [vol/vol] formic acid) and B (99.9% [vol/vol] acetonitrile, 0.1% [vol/vol] formic acid) at 0.50 µl/min to load the peptides (over a 30-min period) and 0.3 µl/min to elute peptides directly into the nano-electrospray; a gradual gradient from 3% (vol/vol) B to 20% (vol/vol) B over 154 min was used, followed by a 12-min fast gradient from 20% (vol/vol) B to 50% (vol/vol) B, at which time a 5-min flash-out from 50 to 95% (vol/vol) B took place. As peptides eluted from the HPLC-column/electrospray source, survey MS scans were acquired in the Orbitrap spectrometer with a resolution of 120,000, followed by MS2 fragmentation of the 20 most intense peptides detected in the MS1 scan from *m/z* 380 to 1,800; redundancy was limited by dynamic exclusion. Raw MS/MS data were converted to MGF file format using msConvert (ProteoWizard [6]) for downstream analysis. The resulting MGF files were used to search against the forward and decoyed-reversed Phialocephala scopiformis protein database via the JGI portal (https://genome.jgi.doe.gov/portal/pages/dynamicOrganismDownload.jsf?organism=Phisc1) with a list of common lab contaminants (available at ftp://ftp.thegpm.org/fasta/cRAP) to establish a false-discovery rate (FDR) (37,222 total entries); the in-house *Mascot* search engine 2.2.07 (Matrix Science) with variable methionine oxidation, asparagine, and glutamine deamidation, plus fixed cysteine carbamidomethylation was used. Scaffold (version 4.7.5; Proteome Software, Inc., Portland, OR) was used for spectral-based quantification and to validate MS/MS peptide and protein identifications. Peptide identifications were accepted if they could be established at greater than 80.0% probability to achieve an FDR of less than 1.0% by the Scaffold local FDR algorithm. Protein identifications were accepted if they could be established at greater than 99.0% probability to achieve an FDR of less than 1.0% and contained at least 2 identified peptides. Protein probabilities were assigned by the ProteinProphet algorithm (7). Proteins that contained similar peptides and that could not be differentiated based on MS/MS analysis alone were grouped to satisfy the principles of parsimony.

**FIG 1:**
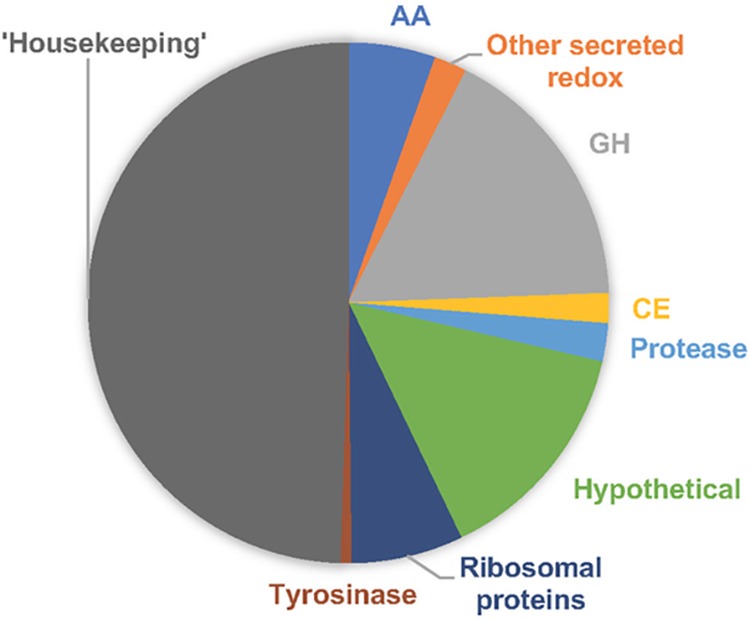
Distribution of 590 *P. scopiformis* proteins detected in medium containing ground lodgepole pine as the sole carbon source. Those categorized as CAZymes included glycoside hydrolases (GHs), auxiliary activities (AAs), and carbohydrate esterases (CEs) and accounted for 24% of the total proteins. Highly expressed ribosomal and housekeeping proteins involved in central metabolism made up 56% of the total. Although function could not be predicted, the 84 sequences classified as hypothetical were generally conserved in other fungi, and 32 sequences featured clear secretion signals. Most (82%) of the identified proteins were present at both time points, and emPAI values (10) were generally highest after 7 days of growth.

Proteins were functionally classified by the top blastp hits among NCBI NR entries, and these were generally consistent with EMBL-EBI predictions of InterPro domains and secretion signals. Carbohydrate-active enzyme (CAZyme) family assignments were made as described previously (8). Consistent with the abundance of transcripts most closely related to P. scopiformis in decayed field samples (1), strain DAOMC 229536 utilizes P. contorta wood as a sole carbon source and produced an array of hydrolytic and oxidative enzymes directly involved in lignocellulose degradation (Fig. 1). Gene expression data are not yet available for related dark septate endophytes (DSEs), but latent saprotrophy may be widespread and merits further investigation.

### Data availability.

The mass spectrometry proteomics data have been deposited to the ProteomeXchange Consortium via the PRIDE (9) partner repository with the data set identifier PXD010720.
